# Advances in Production, Properties and Applications of Sprouted Seeds

**DOI:** 10.3390/foods9060790

**Published:** 2020-06-16

**Authors:** Elena Peñas, Cristina Martínez-Villaluenga

**Affiliations:** Institute of Food Science, Technology and Nutrition (ICTAN-CSIC), Juan de la Cierva 3, 28006 Madrid, Spain; elenape@ictan.csic.es

**Keywords:** seed germination, nutritional value, phytochemicals, bioactivity, health, food safety, technological properties, food development, functional foods

## Abstract

Sprouted grains are widely appreciated food ingredients due to their improved, nutritional, functional, organoleptic and textural properties compared with non-germinated grains. In recent years, sprouting has been explored as a promising green food engineering strategy to improve the nutritional value of grains and the formation of secondary metabolites with potential application in the functional foods, nutraceutical, pharmaceutical and cosmetic markets. However, little attention has been paid to the impact of sprouting on the chemical composition, safety aspects, techno-functional and chemopreventive properties of sprouted seeds and their derived flours and by-products. The six articles included in this Special Issue provide insightful findings on the most recent advances regarding new applications of sprouted seeds or products derived thereof, evaluations of the nutritional value and phytochemical composition of sprouts during production or storage and explorations of their microbiological, bioactive and techno-functional properties.

Sprouted grains, usually designated as a seed with a visible radicle, have been used as food ingredients for many years, based on the general belief they provide significant nutritional, flavor, and textural benefits over non-germinated seed counterparts. In recent years, sprouting has been explored as a promising green food engineering strategy to improve the nutritional value of grains as well as to synthesize secondary metabolites with potential applications in the functional foods, nutraceutical, pharmaceutical and cosmetic markets. In this context, the industry has increasingly launched products containing or made of sprouted seeds. During seed sprouting, a multitude of changes occur, moving from molecular to macroscopic structures. Sprouting reactivates seed metabolism leading to the catabolism and degradation of macronutrients and antinutritional compounds and the biosynthesis of secondary metabolites with potential health benefits. These changes impact the nutritional value and health-promoting potential of the edible seeds. Many researchers around the world have proposed successful strategies such as elicitation to find the optimal environmental conditions during sprout growth able to promote the desired outcomes.

This Special Issue includes six outstanding papers describing examples of the most recent advances in new applications of sprouted seeds or products derived thereof, evaluations of the nutritional value, phytochemical composition and microbiological quality of sprouts during production or storage and explorations of their bioactive and techno-functional properties.

The Special Issue gathers a group of three papers exploring the biochemical composition, nutritional and bioactive properties as well as microbial safety of sprouts obtained from lentil seeds. In this frame, Santos et al. [1] conducted a study aimed at evaluating the protein and mineral profile of sprouts obtained from 12 lentils varieties. Protein, Zn, Mn, Ca and K contents were positively affected by sprouting in most of the lentil varieties studied, suggesting the potential of sprouting technology to improve the nutritional value of legumes. The authors also implemented a disinfection protocol combining SDS reagent and Amukine^®^ application in order to ensure the microbial safety of lentils sprouts without affecting the germination rate and sprout length. Rebollo-Herran et al. [2] focused their research on the evaluation of the impact of lentil sprout intake on the plasmatic levels of melatonin and metabolically related compounds, total phenolic compounds and plasmatic antioxidant status compared to synthetic melatonin. The described results evidence that sprouting enhanced the levels of melatonin, decreasing the content of phenolic acids and flavan-3-ols in lentil. The administration of lentil sprouts to Sprague Dawley rats effectively increased the melatonin levels and antioxidant status in plasma, providing interesting insight on the beneficial effects of lentil sprouting on the attenuation of plasmatic oxidative stress mediated by melatonin. Sikora et al. [3] isolated and characterized polyphenol oxidase isoenzymes (I and II) from stored lentil sprouts and the mechanism of inhibition of these enzymes by antibrowning compounds and cations. The supplementation of sprouts with metal ions (Zn^2+^, Mn^2+^, Fe^3+^) and/or inhibitors (ascorbic acid, citric acid) revealed to be an effective method to decrease the activity of polyphenol oxidase isoenzymes and to prevent enzymatic browning of lentil sprouts during storage and processing.

The Special Issue follows with a short series of articles describing the influence of sprouting on the chemical composition, bioactive and techno-functional properties of cereal flours and milling by-products. In this context, the study of Rico et al. [4] described the optimization of germination conditions to produce barley flour with a superior quality. The results reveal the applicability of sprouting in selected conditions as a valuable strategy to increase the nutritional value, phytochemical content and health benefits of barley. As a result, the authors obtained novel flours from sprouted barley enriched in vitamins, protein, gamma-aminobutyric acid (GABA) and antioxidant compounds. With the same objective, Cardone et al. [5] designed a study aimed at exploring the effect of sprouting on the chemical composition, enzymatic activities, techno-functional properties and bread-making performance of wheat bran, a milling by-product. The obtained results indicate that sprouting triggers positive nutritional changes in wheat bran by decreasing the antinutritional compounds and increasing the fiber content. Techno-functional properties such as water-holding capacity and gluten-aggregation kinetics were also improved as a consequence of the sprouting process, providing valuable characteristics to be used for bread formulation. The authors also demonstrate that breads formulated with wheat bran at a 20% of replacement level are enriched in fiber and show high-quality traits in terms of bread volume and crumb softness. This study evidences that sprouting offers interesting possibilities for the valorization of wheat milling by-products.

Another study conducted by Damazo-Lima et al. [6] reported the chemopreventive potential against colorectal cancer of sprouted oat and its phenolic-avenanthramide extract in a mouse model. Sprouted oat and its phenolic extract are valid chemopreventive ingredients when administered to animals since both reduce inflammation and tumor and adenocarcinoma incidence. Interestingly, sprouted oat exhibited a superior chemopreventive effect over its phenolic extracts, providing experimental evidence for a novel application of sprouted oat as a functional food for colon cancer prevention.

To conclude, the present Special Issue consists of six papers addressing recent advances on the nutritional, bioactive, techno-functional and safety aspects of sprouted grains and their derived flours and by-products, providing insightful information for both the food industry and consumers.

## References

[1] Santos C.S., Silva B., Valente L.M.P., Gruber S., Vasconcelos M.W. (2020). The Effect of Sprouting in Lentil (*Lens culinaris*) Nutritional and Microbiological Profile. Foods.

[2] Rebollo-Hernanz M., Aguilera Y. (2020). Bioavailability of Melatonin from Lentil Sprouts and Its Role in the Plasmatic Antioxidant Status in Rats. Foods.

[3] Sikora M., Swieca M., Franczyk M., Jakubczyk A., Bochnak J., Zlotek U. (2020). Biochemical Properties of Polyphenol Oxidases from Ready-to-Eat Lentil (*Lens Culinaris* Medik.) Sprouts and Factors Affecting Their Activities: A Search for Potent Tools Limiting Enzymatic Browning. Foods.

[4] Rico D., Peñas E., García M.C., Martínez-Villaluenga C., Rai D.K., Birsan R.I., Frias J., Martín-Diana A.B. (2020). Sprouted Barley Flour as a Nutritious and Functional Ingredient. Foods.

[5] Cardone G., D’Incecco P., Casiraghi M.C., Marti A. (2020). Exploiting Milling By-Products in Bread-Making: The Case of Sprouted Wheat. Foods.

[6] Damazo-Lima M., Rosas-Pérez G., Reynoso-Camacho R., Pérez-Ramírez I.F., Rocha-Guzmán N.E., de los Ríos E.A., Ramos-Gomez M. (2020). Chemopreventive Effect of the Germinated Oat and Its Phenolic-AVA Extract in Azoxymethane/Dextran Sulfate Sodium (AOM/DSS) Model of Colon Carcinogenesis in Mice. Foods.

